# Bioethanol production from spent mushroom compost derived from chaff of millet and sorghum

**DOI:** 10.1186/s13068-017-0880-3

**Published:** 2017-08-04

**Authors:** Peter Ryden, Maria-Nefeli Efthymiou, Teddy A. M. Tindyebwa, Adam Elliston, David R. Wilson, Keith W. Waldron, Pradeep K. Malakar

**Affiliations:** 1 The Biorefinery Centre, Quadram Institute Bioscience, Colney, Norwich Research Park, Norwich, NR4 7UA UK; 2 School of Biological Sciences, College of Natural Sciences, Makerere University, P.O. Box 7062, Kampala, Uganda; 3 College of Food Science and Technology, Shanghai Ocean University, 999 Hu Cheng Huan Road, Shanghai, 201306 People’s Republic of China

**Keywords:** *Pleurotus ostreatus*, Lignocellulose, Ethanol, Pre-treatment, Cell walls, Biofuels

## Abstract

**Background:**

In Uganda, the chaff remaining from threshed panicles of millet and sorghum is a low value, lignocellulose-rich agricultural by-product. Currently, it is used as a substrate for the cultivation of edible Oyster mushrooms (*Pleurotus ostreatus*). The aim of this study was to assess the potential to exploit the residual post-harvest compost for saccharification and fermentation to produce ethanol.

**Results:**

Sorghum and millet chaff-derived spent oyster mushroom composts minus large mycelium particles were assessed at small-scale and low substrate concentrations (5% w/v) for optimal severity hydrothermal pre-treatment, enzyme loading and fermentation with robust yeasts to produce ethanol. These conditions were then used as a basis for larger scale assessments with high substrate concentrations (30% w/v). Millet-based compost had a low cellulose content and, at a high substrate concentration, did not liquefy effectively. The ethanol yield was 63.9 g/kg dry matter (DM) of original material with a low concentration (19.6 g/L). Compost derived from sorghum chaff had a higher cellulose content and could be liquefied at high substrate concentration (30% w/v). This enabled selected furfural-resistant yeasts to produce ethanol at up to 186.9 g/kg DM of original material and a concentration of 45.8 g/L.

**Conclusions:**

Spent mushroom compost derived from sorghum chaff has the potential to be an industrially useful substrate for producing second-generation bioethanol. This might be improved further through fractionation and exploitation of hemicellulosic moieties, and possibly the exploitation of the mycelium-containing final residue for animal feed. However, spent compost derived from millet does not provide a suitably high concentration of ethanol to make it industrially attractive. Further research on the difficulty in quantitatively saccharifying cellulose from composted millet chaff and other similar substrates such as rice husk is required.

**Electronic supplementary material:**

The online version of this article (doi:10.1186/s13068-017-0880-3) contains supplementary material, which is available to authorized users.

## Background

There is increasing pressure to optimise the use of agricultural by-products for reasons of environmental and economic sustainability. In intensive cereal growing areas in Africa, residual straw is an abundant by-product and is used as a substrate for mushroom cultivation [1] and research on utilisation of sorghum wastes has demonstrated successful use of the straw as a substrate for *Pleurotus* cultivation [2]. There is also much interest in utilising it as a feedstock for second-generation (2G) biofuel production as evidenced by studies on pre-treatment and saccharification of sorghum straw and bagasse from juice-extracted sweet sorghum [3, 4], and also its pre-treatment, saccharification and fermentation [5–8].

However, 2G biofuel production from lignocellulose faces many challenges, both technical and economic. Hydrothermal/chemical pre-treatments are expensive, and saccharification and fermentation at high substrate concentration requires considerable capital investment. Also of importance is the limited availability and/or prohibitive cost of the feedstock in areas where there are well-established alternative local markets.

In Uganda, millet and sorghum straw produced on marginal semi-arid land are widely used as animal fodder so are not regarded as waste. However, Oyster mushrooms (*Pleurotus ostreatus*) are cultivated widely in Uganda on substrates derived from the chaff remaining from threshed panicles of millet and sorghum. Indeed chaff, as the least valuable waste stream, has been used for commercial *Pleurotus* cultivation in south western Uganda since 1995. Since 2007, the Mushroom Training and Resource Centre (MTRC; http://www.oystermushroom.ug/) has coordinated the training and marketing of *Pleurotus* production in the region. The chaff is soaked in water for 3 days, left to ferment, sterilised in a fire-heated steel drum whilst wrapped in banana leaves, bagged and inoculated. After several flushes, the mushroom compost is discarded.

The spent mushroom compost may be considered as an interesting lignocellulosic substrate for second-generation biofuel production because it will have undergone partial degradation by the Oyster mushroom mycelium. *Pleurotus ostreatus* is a white rot fungus with peroxidase and laccase activities [9, 10] and the partial degradation of the lignocellulose might be expected to decrease the pre-treatment severity needed for saccharification. Such an effect has been observed in pre-treatments with ammonia fibre expansion of rice straw lignocellulose before and after *Pleurotus* cultivation [11].

Elliston et al. [12] developed state-of-the-art high-throughput methods for the screening of lignocellulose for saccharification to glucose and fermentation to ethanol. In this approach, the biomass is milled to a powder, hydrothermally pre-treated, and then the saccharification and fermentation is carried out with excess enzyme and at up to 10% (w/v) substrate concentration. These methods have been found to give a good indication of yields at larger scales using higher substrate concentrations of un-milled biomass steam exploded at the same severities.

The aim of this research has been (1) to employ high-throughput screening methods to study and compare the conversion of mushroom compost lignocellulose from sorghum and millet chaff, to identify the likely optimal pre-treatment and saccharification conditions (particularly enzyme loadings), and suitable inhibitor-resistant yeasts for fermentation of sugars to ethanol, and (2) to carry out simultaneous saccharification and fermentation to facilitate SSF at higher substrate concentrations, thereby maximising the concentration of ethanol produced in order to reduce downstream distillation costs and confirming optimal enzyme concentrations.

## Methods

### Biomass

The spent mushroom compost was supplied by the Mushroom Training and Resource Centre (MTRC), Kyanamira, Kabale, Uganda. On delivery, the bags were opened in a MSC Class 2 cabinet and the samples were transferred to 2.5, 5 or 10 L polypropylene buckets and contained in autoclave bags. Sterilisation proceeded at 127 °C for 20 min. The moisture content of the sterilised composts was measured after drying duplicate samples at 60 °C for 66 h. The stones and the larger lumps of mycelium were removed from the samples but smaller particles of mycelium were retained.

### Neutral sugar analysis

Triplicate samples of 2–4 mg of milled material (<0.5 mm Retsch cyclone mill Twister, Retsch Ltd. UK) were dispersed in 65 μL 72% w/w H_2_SO_4_ at room temperature for 3 h then diluted to 1 M with 715 μL water and heated at 100 °C for 2.5 h then cooled on ice. 200 μL of 1.00 mg m/L 2-deoxy glucose was added. The whole sample was reduced, acetylated and quantified by gas chromatography (GC) as described [13].

### Uronic acid analysis

Triplicate samples of 3–8 mg of milled material were dispersed in 200 μL 72% w/w H_2_SO_4_ at room temperature for 3 h then diluted to 1 M with 2.2 mL water and heated at 100 °C for 1 h then cooled on ice. 2.6 mL of water was added, and then the samples were filtered through a glass fibre filter (GF/C) into 5-mL Eppendorf tubes and frozen. Uronic acids were measured with a glucuronic acid standard using volumes of 1.8 mL Na_2_B_4_O_7_/c.H_2_SO_4_, 0.3 mL of sample solution and 30 μL of 0.15% 3-phenyl phenol in 0.5% NaOH [14]. After storage in the dark for 30 min, absorbances were measured at 520 nm.

### Small-scale hydrothermal pre-treatments

Small-scale experiments to find a suitable pre-treatment severity employed microwave irradiation (Biotage Initiator^+^, Uppsala, Sweden) on samples Retsch-milled to <0.5 mm. The dry weights were measured after drying at 60 °C for 16 h. Samples containing 250 mg dry weight were placed in 10-mL-thick-walled glass tubes with a small Polytetrafluoroethylene (PTFE)-coated stirrer bar. Water was added to bring the total to 5 mL. The tubes were crimp-capped with silicone/PTFE septa. The combined effects of time and temperature of steam treatment are described by a severity factor [15] defined by Eq. 1:

1 $$\text{Severity}\,\text{factor} = \log_{10} (t \times \exp ((T - 100)/14.75)).$$

Severity factors ranging from 3.00 to 4.75 are shown as hyperbolas in Additional file 1: Figure S1. A typical test for a range of severities by steam explosion on straw or woody samples might use 10-min treatments from 170 to 230 °C. In the microwave-powered method, temperatures were limited to 208 °C so as not to exceed the maximum pressure that the glass tubes can withstand but conditions of equivalent severity can be calculated (Additional file 1: Figure S1).

### Large-scale steam explosion pre-treatments

One batch of each of the spent mushroom compost samples was steam exploded using a Cambi™ steam explosion pilot plant [16]. Only a small amount of KAN03 was available so 238 g was steam exploded; for all other samples 500 g was used. Warm water (50 °C) was added to the chamber (4.3 L/kg dry mass). Millet samples were steam treated for 10 min at 5.2 bar (160 °C, severity factor 2.77) and the sorghum samples were steam treated for 10 min at 14.5 bar (200 °C, severity factor 3.94). The steam-exploded slurries were centrifuged at 4200 rpm for 20 min. The dry matter in the supernatants was measured by drying samples at 40 °C for 16 h. The dry matter in the pellets was measured after drying at 40 °C for 5 days.

### Saccharification

The microwave-pre-treated slurries were centrifuged in 13-mL Falcon tubes at 3150*g* for 20 min and the pellets were washed twice with water. Saccharification was performed at 50 °C for 96 h with excess enzyme at 5% (w/v) substrate concentration in a volume of 5 mL. This involved adding the following volumes to the wet pellets: 2.5 mL 0.2 M NaOAc pH 5.0, 0.1 mL (5 mg/mL) thiomersal, 1.44 mL water, 50 μL Cellic CTec2 (30 FPU/g) and 5 μL Cellic HTec2 (Novozyme).

The tubes were centrifuged at 3500 rpm for 20 min and the supernatant was poured into 5-mL Eppendorf tubes. The supernatants were diluted as follows: 0.1 mL sample + 0.9 mL water for the sorghum samples and 0.2 mL sample + 0.8 mL water for the millet samples.

Glucose monosaccharide was measured with a Megazyme kit (d-Glucose Assay Kit GOPOD Format) and a 10 mM glucose standard in 96-well plates using 3, 6 and 10 μL of sample and 7, 4 and 0 μL water. GOPOD solution (0.3 mL) was added to each well and the plate was heated at 50 °C for 20 min. Absorbance was measured at 505 nm.

Xylose monosaccharide was measured with a Megazyme kit (d-Xylose assay kit) and a 0.25 g/L xylose standard in 96-well plates using 10 μL of sample and 0.282 mL of a mixture of 12 mL water, 2.4 mL buffer, 2.4 mL NAD^+^ plus ATP and 0.12 mL hexokinase. The absorbance at 340 nm was read after 4 min then 5 μL of xylose mutarotase + xylose dehydrogenase was added. The absorbance was read when the reaction had gone to completion (about 20 min).

### Enzyme optimisation

The compost samples with the highest cellulose content for millet and sorghum were microwave irradiated at severity factors of 2.77 for millet (10 min at 160 °C) and 3.94 for sorghum (10 min at 200 °C). Bulk samples were prepared; 5 × 750 mg biomass + 13 mL water in 50 mL tubes. The pre-treated material was transferred to Falcon tubes, washed twice with water and centrifuged at 3150*g* for 20 min. The wet pellets were divided into 13-mL Falcon tubes to test the amount of enzyme needed with 5% substrate concentration calculated from the initial dry matter, at pH 5.0 in 0.1 M NaOAc and 0.1 g/L thiomersal in 18-h experiments at 50 °C.

### Simultaneous saccharification and fermentation (SSF)

A small-scale SSF experiment was performed in a 96-well plate format in 1 mL volumes to screen the capabilities of six yeast strains against the three millet composts and four sorghum pre-treated composts at a substrate concentration of 2.5% (w/v). The millet and sorghum composts were milled to <0.5 mm and 750 mg dry mass was microwave treated for 10 min in 13 mL water at 160 and 200 °C, respectively. The pre-treated samples were centrifuged for 20 min in 15-mL Falcon tubes at 3150*g*. The pellet volumes were approximately 2 mL. The pellets were washed twice with water. The pellet moisture contents were measured in duplicate by drying small samples for 16 h at 40 °C. The supernatants were retained and the mass of solids in the supernatants was measured in triplicate on a drying balance at 105 °C to the nearest mg.

1-mL sterile 2D barcoded polypropylene tubes with screw caps in 96-well racks were obtained from Thermo Scientific (Tube TrakMates; 2D barcoded storage 1.0-mL tube screw top sterile polypropylene with caps latch rack, Thermo Scientific Matrix). Wet pellet samples (25 mg dry mass) were weighed into the tubes and two 2.5-mm glass beads were added. Water was added to bring the water content of all tubes to 100 μL after which they were autoclaved. All other additions; enzymes, yeast and yeast nitrogen base (YNB), were combined into 15 mL stock solutions.

YNB was obtained from Formedium™ (Hunstanton, CYN0201). Two concentrations were prepared, 6.9 g/L for culturing the yeasts and rinsing the pellets and 7.67 g/L for the final rinse and making up the yeast + enzyme sample to 0.9 mL to be added to the wet pellets. The solutions of yeast nitrogen base were autoclaved.

The 6 yeasts used included 5 furfural-resistant strains: *S. paradoxus*: NCYC 3277, and *S. cerevisiae*: NCYC 3312, NCYC 3290, NCYC 3284 and NCYC 3451. In addition, NCYC 2826 which has high ethanol tolerance was also included. Yeast strains were cultured over a weekend in 10 mL YNB + sugar. These cultures were centrifuged at 3000 rpm for 5 min. The supernatants were decanted. The pellet was washed twice with YNB then with YNB at 1.11 times concentration and made up to 15 mL. 30 μL CTec2 (12 FPU/g biomass) and 3 μL HTec2 were added to each yeast suspension. The biomass samples were arrayed by rows and the yeast cultures by columns as shown (Additional file 2: Figure S2).

The rack was set on its side on a rotary plate so that the tubes were horizontal and incubated at 25 °C for 72 h at 120 rpm. Then the rack was put in a boiling water bath for 10 min. After cooling, the rack was centrifuged at 3000 rpm. The supernatants were individually filtered [4 mm syringe filters, 0.45 μm Polyvinylidene fluoride (PVDF) membrane] into GC vials for high-performance liquid chromatography (HPLC). Ethanol standards, 0.3, 0.5, 0.8, 1.0 and 1.3% v/v, were prepared.

### SSF at 30% substrate concentration

Larger scale SSF experiments were carried out with two compost samples: the highest ethanol-yielding millet and sorghum composts which also had the highest glucose compositions, KAN01 and KAB08. The steam-exploded and centrifuged pellets contained too much water to be fermented at 30% substrate concentration so some water was removed through 10 μm nylon bolting cloth to make the dry/wet ratio high enough (KAN01 0.3598 g dry/g wet; KAB08 0.3816 g dry/g wet).

Wet steam-exploded biomass samples were saccharified and fermented at 30% substrate concentration in 10 mL volumes (3 g dry matter) in 49 mL plastic pots (Securitainer with tamper evident push on cap, Ampulla Limited, Cheshire, SK14 2NY, UK), and water was added. A 25.4-mm-diameter ceramic ball (2.25 L Porcelain ball charge. Capco Test Equipment, Ipswich, Suffolk, IP1 5AP, UK) was placed in the pot and the pellet of biomass + water was compressed by the ball and formed into a bowl-shaped depression. The purpose of this is so that when the yeast and enzymes are added, fermentation proceeds from the surface with an initially low substrate concentration.

The pots were warmed to 37 °C. Triplicate samples at 3 enzyme levels, 10, 15 and 20 FPU/g, and with two yeast cultures NCYC 2826 and NCYC 3312 were prepared. The yeast cultures had been grown up over 3 days from slopes, in yeast medium (YM DIFCO) at 25 °C. The cultures were centrifuged at 2000 rpm for 5 min. The pellets were washed twice with yeast nitrogen base, and then made up in 2.9 times YNB. The yeast suspensions were combined with the enzymes and added to the pots. The pots were capped. A 0.5 mm bore 25-mm-length needle was put into each pot with a cotton wool plug to let CO_2_ escape. The pots were weighed, and then incubated at 37 °C and 150 rpm for 96 h. There were 6 blanks with yeasts and enzymes. The fermented material was centrifuged in 13-mL Falcon tubes at 3150*g* for 20 min. The supernatants were centrifuged again in Eppendorf tubes at 10,000*g* for 10 min. These second supernatants were filtered individually through 0.4 μm syringe filters. The filtered samples were sealed in GC vials and analysed for glucose and ethanol by HPLC using a Flexar_ FX-10 UHPLC instrument (Perkin Elmer, UK) equipped with a refractive index detector and an Aminex HPX-87H organic acid analysis column (Bio-Rad Laboratories Ltd., UK; 65 °C, mobile phase 5 mM H_2_SO_4_, flow rate 0.6 mL/min).

## Results and discussion

### Biomass sources and characterisation

Spent compost from millet and sorghum chaff were sourced from 2 districts in the Kigezi sub-region of Western Uganda; Kanungu (sampling date 09/09/2014) and Kabale (sampling date 11/09/2014), see Table 1.

**Table 1:** Biomass Samples, location of origin (district), quantities and moisture contents

District	Sample name	Biomass	Fresh mass (g)	Moisture (%)
Kanungu	KAN01	Millet	1356	13.1
Kanungu	KAN02	Millet	1652	10.3
Kanungu	KAN03	Millet	511	8.2
Kanungu	KAN04	Sorghum	5319	9.4
Kabale	KAB06	Sorghum	6084	9.9
Kabale	KAB07	Sorghum	5750	9.5
Kabale	KAB08	Sorghum	2068	14.7

All of the composts contained white lumps of mycelium, which were removed with a 4-mm sieve. One millet sample (KAN03) contained some stones >6 mm, snail shells and insect cuticles; these were also removed with the 4 mm sieve.

### Polysaccharide analysis

The plant polysaccharide compositions in the spent composts, determined by acid hydrolysis of milled material, are shown in Table 2. The glucosamine content was not measured since the yeasts used cannot ferment amino sugars from the fungal mycelium. The highest cellulose content of the spent mushroom composts was 12% for compost derived from millet, and 18% for compost derived from sorghum. The cellulose levels are much lower than the 30% cellulose content of rice straw after the 3rd flush (48 days) of *Pleurotus* cultivation [17]. For comparison, the cellulose content of biomass without mushroom cultivation is sorghum straw (32% [4]) and sweet sorghum bagasse (42 [5]; 40% [6]).

**Table 2:** Monosaccharide compositions (g/kg) of the plant cell wall polysaccharides in the spent mushroom composts, hydrothermally pre-treated (microwave), and steam exploded residues (*n* = 3)

Means (sd)								
Sample	Rha	Fuc	Ara	Xyl	Man	Gal	Glc	UA
Spent composts								
KAN01	4.3 (0.1)	2.9 (0.1)	44.6 (1.7)	64.6 (2.3)	11.0 (0.2)	20.1 (0.6)	108.4 (0.4)	43.7 (2.4)
KAN02	4.5 (0.1)	3.3 (0.0)	45.0 (0.2)	63.0 (0.8)	10.3 (0.2)	21.0 (0.3)	115.0 (1.5)	44.8 (4.5)
KAN03	3.0 (0.5)	1.3 (0.3)	19.6 (2.2)	41.6 (8.2)	5.5 (0.9)	9.4 (1.7)	83.8 (1.6)	29.3 (3.1)
KAN04	2.1 (0.3)	0.7 (0.1)	17.3 (3.2)	114.8 (17.0)	3.9 (0.6)	4.7 (0.8)	142.3 (18.6)	21.0 (1.6)
KAB06	2.0 (0.1)	0.6 (0.1)	17.9 (1.4)	166.0 (9.8)	3.6 (0.6)	4.7 (0.4)	181.1 (9.9)	21.7 (1.2)
KAB07	2.1 (0.2)	0.7 (0.1)	16.7 (1.2)	131.0 (8.5)	4.3 (0.5)	4.5 (0.5)	163.0 (2.2)	22.4 (1.2)
KAB08	2.1 (0.5)	0.6 (0.1)	16.9 (1.4)	139.2 (0.4)	3.8 (0.8)	5.1 (0.8)	157.8 (7.3)	25.2 (3.4)
Microwave pre-treated								
KAN01	1.8 (0.6)	1.4 (0.2)	32.8 (5.2)	58.7 (9.5)	3.3 (0.5)	10.6 (9.1)	135.0 (21.3)	nd
KAN02	1.7 (0.1)	1.4 (0.1)	21.6 (0.8)	39.8 (1.9)	3.2 (0.0)	10.7 (0.5)	123.8 (5.1)	nd
KAN03	1.8 (0.1)	0.8 (0.0)	15.9 (0.3)	33.0 (0.4)	2.9 (0.0)	7.4 (0.0)	87.3 (3.8)	nd
KAN04	1.1 (0.4)	0.0 (0.0)	5.2 (2.7)	31.1 (0.7)	0.8 (0.1)	0.6 (0.3)	146.2 (21.4)	nd
KAB06	1.0 (0.1)	0.0 (0.0)	2.4 (0.2)	42.1 (0.5)	1.1 (0.5)	0.6 (0.0)	193.0 (0.8)	nd
KAB07	0.7 (0.1)	0.1 (0.0)	2.3 (0.2)	29.6 (0.6)	0.8 (0.0)	0.6 (0.0)	128.3 (3.9)	nd
KAB08	1.4 (0.1)	0.1 (0.1)	4.7 (0.2)	64.0 (1.0)	2.0 (0.2)	1.3 (0.1)	262.4 (5.7)	nd
Steam exploded								
KAN01	2.8 (0.0)	1.7 (0.0)	30.3 (1.0)	55.9 (1.7)	6.2 (0.1)	14.2 (0.4)	146.0 (2.9)	30.0 (4.5)
KAN02	2.5 (0.2)	1.8 (0.2)	21.9 (0.6)	37.9 (1.0)	5.2 (0.1)	10.8 (0.3)	115.5 (2.9)	22.9 (5.2)
KAN03	2.5 (0.1)	1.3 (0.1)	16.7 (0.1)	36.9 (0.5)	3.8 (0.1)	8.6 (0.2)	111.4 (1.4)	21.2 (1.3)
KAN04	1.3 (0.1)	0.1 (0.0)	4.5 (0.1)	46.9 (1.4)	1.6 (0.0)	1.4 (0.0)	171.6 (4.4)	7.1 (2.0)
KAB06	1.2 (0.2)	0.1 (0.0)	4.5 (0.8)	50.3 (7.4)	1.5 (0.1)	1.4 (0.2)	159.0 (20.8)	6.4 (1.2)
KAB07	1.2 (0.0)	0.1 (0.0)	4.6 (0.2)	50.7 (0.2)	1.6 (0.0)	1.5 (0.1)	158.5 (1.8)	8.6 (1.2)
KAB08	2.2 (0.3)	0.3 (0.0)	6.5 (0.2)	78.5 (2.7)	2.5 (0.2)	2.7 (0.1)	253.7 (17.7)	7.9 (2.4)

Millet: samples KAN01-3; Sorghum: samples KAN04-8. *Rha* Rhamnose, *Fuc* Fucose, *Ara* Arabinose, *Xyl* Xylose, *Man* Mannose, *Gal* Galactose, *Glc* Glucose, *UA*, Uronic acid

### Microwave hydrothermal pre-treatment

The microwave-powered hydrothermal pre-treatment assessments were all carried out at 208 °C to ensure equal areas under the curves during the heating and cooling periods at all severities (Additional file 3: Figure S3).

**Fig. 1:**
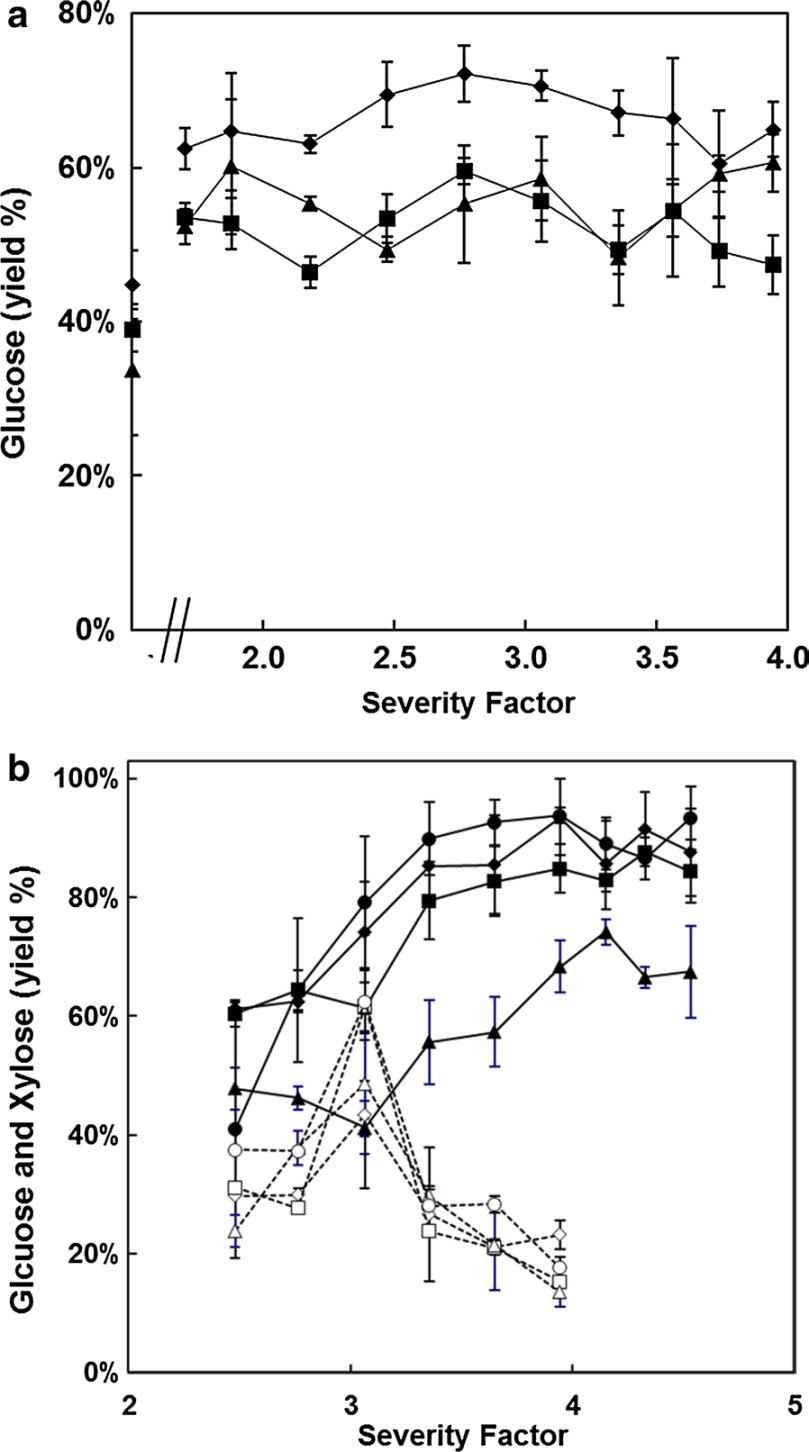
**a** Mean and standard deviation (g/100 g) polymeric glucose in the original material released by cellulase and hemicellulases (30 FPU/g) from triplicate samples of spent mushroom compost derived from millet; ◆ KAN01, ■ KAN02, ▲ KAN03. The data points on the ordinate axis comprise samples without microwave pre-treatment. Xylose hydrolysis was not measured. **b** Average and standard deviation (g/100 g) polymeric glucose and xylose in the original material released by cellulase and hemicellulases from triplicate samples of spent mushroom compost derived from sorghum; filled symbols glucose, open symbols xylose: ◆◊ KAN04, ■□ KAB06, ▲△ KAB07, ●○ KAB08

The optimum pre-treatment conditions for saccharification of millet and sorghum composts were determined in triplicate (separate pre-treated samples) using excess cellulase. The saccharification results are shown for millet (Fig. 1a) and sorghum (Fig. 1b). The profiles are very different. For millet, the response to severity was essentially flat, from the lowest severity tested (1.703) to the highest (nearly 4.0). Maximum saccharification of between 60 and 70% was achieved. Nevertheless, pre-treatment enhanced saccharification potential compared with the non-pre-treated material. In contrast, sorghum showed a very different response curve. Severities >3.355 released most glucose, and for samples KAN04 and KAB08, this was close to 100%. The preferable severity factors for millet and sorghum were 2.77 and 3.94, respectively. Since sorghum composts contain appreciable amounts of xylose-containing polymers (Table 2), the release of xylose was also assessed (Fig. 1b). In keeping with other studies on lignocellulose [18], much of this degrades during pre-treatment (Fig. 1b). This degradation is likely to render the cellulose more accessible to cellulases, but it also creates fermentation inhibitors such as 2-furfural.

### Enzyme optimisation

**Fig. 2:**
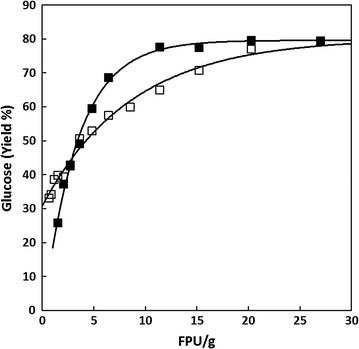
Millet (□ KAN01) and sorghum (■ KAB06) saccharification with varying amounts of cellulase. *N* = 1; (g/100 g) polymeric glucose in the original material released by cellulase

Enzyme optimisation was performed using optimally pre-treated millet (KAN01) and sorghum (KAB06), as shown in Fig. 2. The results showed that enzyme loading in the region of 12 FPU/g substrate or more was suitable.

### SSF of milled and hydrothermally pre-treated spent composts

The polysaccharide compositions of the pellets from milled millet and sorghum, microwave treated for 10 min at 160 and 200 °C (severities of 2.77 and 3.94), respectively, were determined (Table 2). The results of the analysis show that 1) similarity in the respective compositions of composts from sorghum or millet chaffs; 2) the microwave hydrothermal pre-treatment has changed the chemistry of sorghum and millet composted lignocellulose significantly. In both cases, the level of many of the non-cellulosic sugars, particularly xylose and arabinose, were much reduced. In sorghum, xylose, for example, had been reduced by approximately 70%. In millet, which had contained relatively less xylose in the original mushroom compost, it was reduced by approximately 15%. These losses will have been due to the autohydrolysis of the non-cellulosic polysaccharides under the pre-treatment conditions.

**Fig. 3:**
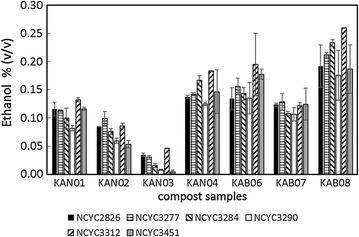
A bar chart of ethanol concentration from each microwave-pre-treated compost sample in small-scale SSF experiments with six yeast strains. SSF was carried out in duplicate for 72 h at 25 °C at a substrate concentration of 2.5% (w/v) and a cellulase loading of 12 FPU/g biomass. See figure for shading regime

Small-scale SSF was performed in a 96-well plate format in 1 mL volumes to screen the fermentation capabilities of six selected yeast strains against the pre-treated millet and sorghum composts. In order to optimise the fermentation, the range of yeasts included high ethanol-yielding, and furfural-resistant strains identified previously [19] with tolerance to stresses created during biorefining of lignocellulose [20]. The final concentrations of ethanol in each well are shown (Fig. 3). Interestingly, the results showed considerable variation on the basis of yeast and biomass source. The most effective yeast was NCYC 3312 for all substrates, whilst KAN01 and KAB08 were the highest yielding of the millet and sorghum composts. Broadly, the yield of ethanol (Fig. 3) generally followed the level of (cellulosic) glucose present in the pre-treated lignocellulose (Table 2).

### Steam explosion

Small pilot-scale pre-treatment experiments employed steam explosion [21]. All 3 millet-derived composts were pre-treated at a severity factor that was the optimum for the sample that gave the highest glucose yield in the previous saccharification experiments (2.77, 160 °C 10 min); likewise for the 4 sorghum-derived composts (3.94, 200 °C 10 min).

The recovery of steam exploded slurry was not quantitative due to the characteristics of the apparatus, and some material (circa 10%) was lost through venting. The recovery of total mass and the distribution of that mass between supernatant and pellet is shown in Table 3.

**Table 3:** The  % recoveries of each substrate after steam explosion and its distribution between supernatant and pellet

	Starting dry matter (g)	Recovery (%)	Solids distribution	
			Supernatant (%)	Pellet (%)
KAN01	451.9	89.2	34.5	65.5
KAN02	451.7	93.8	36.2	63.8
KAN03	219.1	96.9	23.7	76.3
KAN04	466.6	91.4	22.4	77.6
KAB06	483.8	87.0	21.7	78.3
KAB07	469.6	91.7	22.1	77.9
KAB08	474.9	92.0	29.1	70.9

The compositions of the steam-exploded pellets were determined (Table 2). Changes in chemical composition of the lignocellulosic residue by the pre-treatment were similar to those that occurred during the small-scale hydrothermal pre-treatment (Table 2).

### SSF at 30% (w/v) substrate concentration

**Fig. 4:**
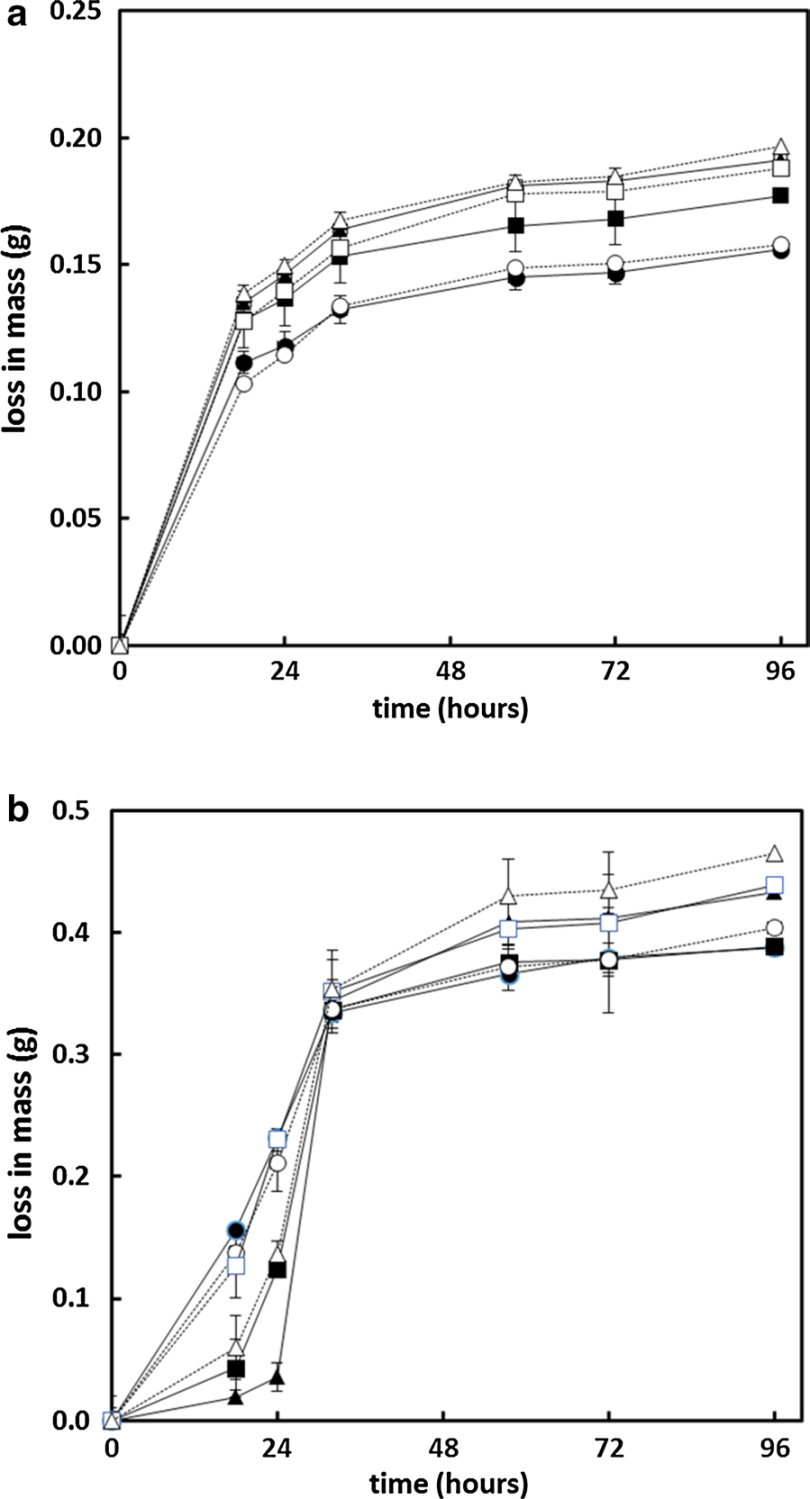
**a** SSF of steam-exploded millet compost (KAN01) at 30% (w/v) substrate concentration and 3 cellulase levels: ●○ 10 FPU/g, ■□ 15 FPU/g, ▲△ 20 FPU/g substrate. *Open symbols* NCYC 2826, *closed symbols* NCYC 3312. *N* = 3. **b** SSF of steam-exploded sorghum compost (KAN08) at 30% substrate concentration and 3 cellulase levels: ●○ 10 FPU/g, ■□ 15 FPU/g, ▲△ 20 FPU/g. *Open symbols* NCYC 2826, *closed symbols* NCYC 3312. *N* = 3

Using steam exploded residues, larger scale (10 mL) SSF experiments at industrially relevant substrate concentrations were carried out with the highest yielding of the millet and sorghum composts which also had the highest glucose compositions: KAN01 and KAB08. These two samples were saccharified and fermented at 30% (w/v) substrate concentration. In order to achieve the high substrate concentrations, some of the water was removed by pressing against 10 μm nylon bolting cloth (KAN01 0.3598 g dry/g wet; KAB08 0.3816 g dry/g wet). An indication of the progress of the fermentation and when it had reached a plateau was obtained by measuring the loss in predominantly CO_2_ mass (Fig. 4).

**Fig. 5:**
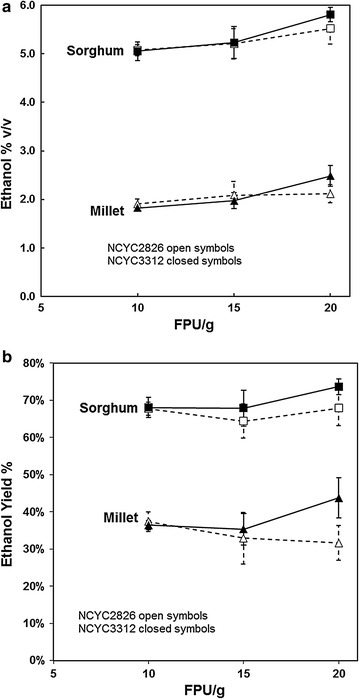
Ethanol concentration after 96 h SSF at 30% substrate concentration. ■□ sorghum, ▲△ millet. *Open symbols* NCYC 2826, *closed symbols* NCYC 3312. Averages of triplicate ± standard deviation. **a** Ethanol concentration, (% v/v); (**b**) ethanol yield,  % theoretical maximum assuming all the cellulose in the steam-exploded material is fermented with the stoichiometry of 1 Glucose 2 Ethanol

After about 30 h of SSF, a clear difference was observed between sorghum and millet fermentations in keeping with the earlier assessments (Fig. 4). After 96 h, it was observed that the sorghum samples had liquefied but the millet had not. The ethanol concentrations achieved after 96-h fermentation were much higher for sorghum than for millet (Fig. 5). Of the two yeasts assessed, strain NCYC 3312 gave only a slightly higher ethanol yield than NCYC 2826. Under these conditions, the ethanol concentration achieved from sorghum was 45.8 g/L (5.81% v/v; Fig. 5a) which is in the order of the level required for industrial distillation (≥50 g/L [21, 22]). Use of high torque reactors, slightly higher substrate concentrations and optimisation of SSF conditions to increase the yield (which was in the region of 65–70% for Sorghum) might be expected to achieve this. Nevertheless, variability in the levels of cellulose in the different samples of sorghum-derived mushroom compost will need to be addressed. This and other properties of the spent compost may be related to the mushroom yield. Restricting the time of *Pleurotus* cultivation can minimise the loss of cellulose [11]. Thus, taking the compost after 2 flushes may be better than after three, although this would be likely to have a negative impact on the economics of mushroom production.

**Fig. 6:**
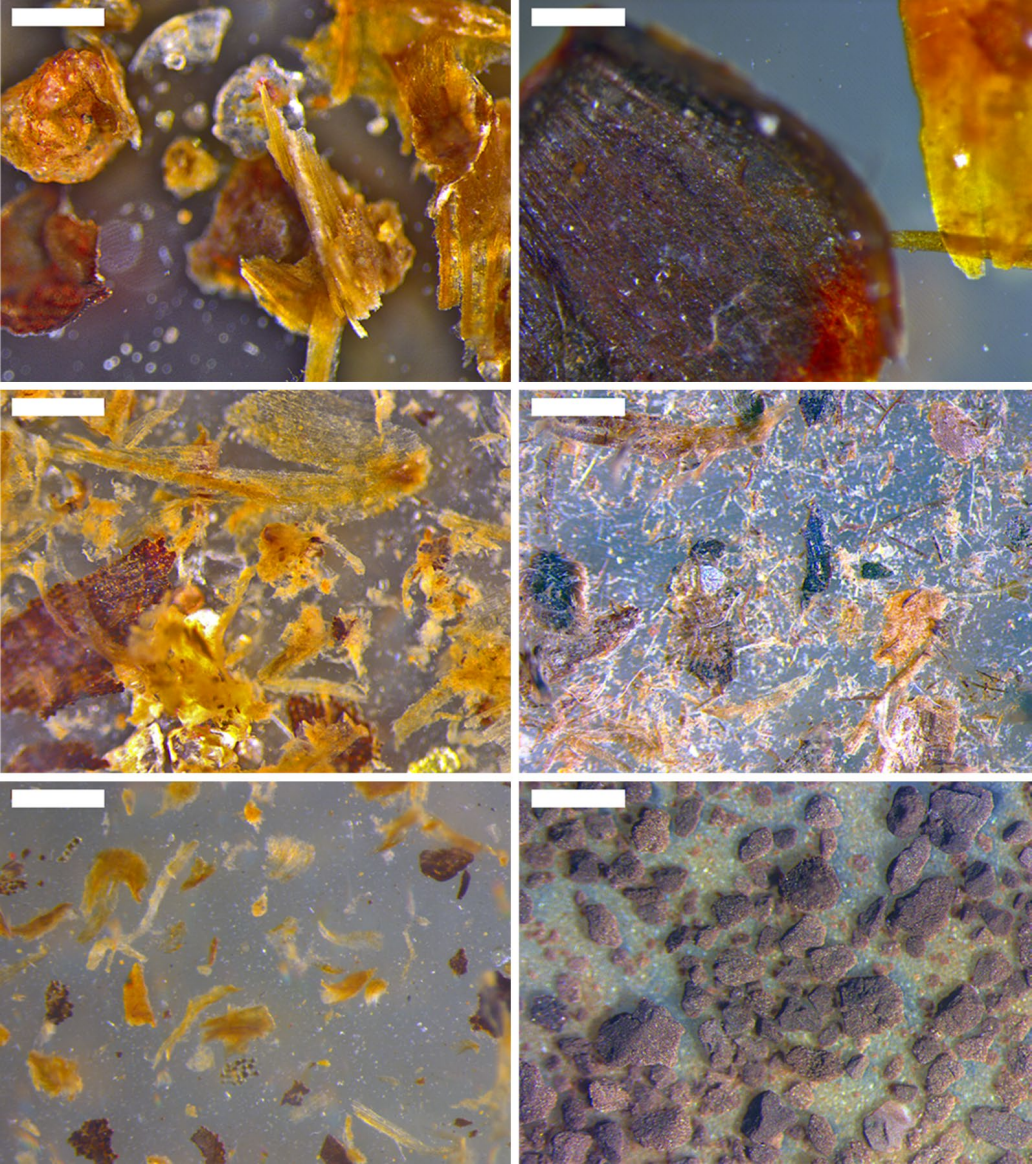
Light micrographs of millet (*left*) and sorghum (*right*) composted material before pre-treatment (*top*), after steam explosion (*middle*) and after SSF (*bottom*). *Scale bars* 0.5 mm

The visual appearance of representative samples of millet and sorghum composted material is shown in light micrographs before and after steam explosion, and after SSF (Fig. 6). The micrographs show clearly the enhanced fragmentation of the materials after steam explosion pre-treatment, reflecting the breakdown of the tissues through fracture and cell separation as found in wheat bran [23] and dissolution of some of the cell wall components. Much of the finer material appears to have been lost after SSF. However, the bulk of larger particulate material remains.

Notwithstanding the lower levels of cellulosic material in Millet chaff-derived compost, the microscopy and chemical analyses carried out on the samples fail to indicate why the millet-substrate was so much poorer than sorghum chaff-derived material in terms of ethanol yield. Both plants are monocotyledonous, and have similar classes of cell wall components. However, there may be differences in the nature of the lignin and possibly other cell wall components such as silica. Recently, Wood et al. [18] showed that rice husk, although similar in carbohydrate composition to rice straw, was much less amenable to saccharification and fermentation after hydrothermal pre-treatment. It may be possible through breeding strategies to improve the properties of millet chaff, and this may also impact on mushroom production also.

For the future, as the Ugandan (and other African) mushroom cultivation industries develop, and move from essentially cottage industries to more sophisticated industrial scales, large localised co-product streams from e.g. spent sorghum and millet chaff mushroom compost are likely to be created. The current work has demonstrated that there is indeed potential to exploit these residues for producing bioethanol. Continued development of lignocellulose industrial biotechnology will augment such strategies. For example, the development of yeasts that can create much more valuable platform and fine chemicals would improve the economic sustainability of residue utilisation. Furthermore, the potential to exploit the non-cellulosic components should not be ignored. Recent research has demonstrated the potential to recover anti-oxidants from wheat chaff [24] including the use of ultrasound-assisted extraction [25]. Such fractionation could be incorporated into the pre-treatment process step, resulting in several value-added products. Final residues, which could still include residual protein from the mycelium, might provide ingredients for animal feed. There has already been significant research to create ruminant feed ingredients from fungal-treated lignocellulosic biomass as reviewed by Kuijk et al. [26].

Although not considered in this study, *P. ostreatus* contains arabitol, pleuran (a β-glucan) and chitin in its mycelium [27]. No attempt was made to saccharify the chitin since it would not be compatible with *Saccharomyces* fermentation. Glucosamine inhibits *Saccharomyces*. Yeasts do exist which can assimilate glucosamine [28] and *Mucor circinelloides* can ferment chitin to ethanol [29] and this may provide further technological opportunities to exploit mushroom composts.

## Conclusions

Three mushroom compost samples derived from millet chaff and four from sorghum chaff have been evaluated for their compositions, and propensity for saccharification and fermentation to ethanol. Millet chaff-derived compost was unsuitable and failed to produce a substantial yield even at high substrate concentration. However, sorghum chaff-derived compost, after optimisation of hydrothermal pre-treatment and saccharification, provided a good substrate for SSF. At 30% (w/v) substrate concentration, it liquefied effectively and yielded ethanol at a concentration of 5.81% (v/v) (4.8% w/v). With further improvements in % yield (circa 70%) and maybe increased substrate concentration through batch addition, it should be possible to increase the ethanol yields further.

Of the 5 furfural-resistant yeast strains tested, one strain (NCYC 3312) provided higher ethanol concentrations than a Spanish wine strain (NCYC 2826) in small-scale experiments at low substrate concentration, but showed only a slight improvement over NCYC 2826 at high substrate concentrations.

## Additional files

**Additional file 1.** Pre-treatments at equivalent severities for steam treatments for 10 min and microwave treatments at 208 °C.

**Additional file 2.** Table showing sample layout on plates.

**Additional file 3.** Temperature during short (bold trace) and long microwave treatments. The curves are coincident up to 1.81 min.

## Abbreviations

2G: second generation
NCYC: National Collection of Yeast Cultures (UK)
MTRC: Mushroom Training and Resource Centre
GC: gas chromatography
HPLC: high-performance liquid chromatography
PTFE: polytetrafluoroethylene
NAD: nicotinamide adenine dinucleotide
ATP: adenosine triphosphate
YNB: yeast nitrogen base
SSF: simultaneous saccharification and fermentation
PVDF: polyvinylidene fluoride
