# Supplementary figures and images for: Bioethanol production from spent mushroom compost derived from chaff of millet and sorghum
Source: Biotechnol Biofuels. 2017 Aug 4;10:195. doi: 10.1186/s13068-017-0880-3 (PMC5545022; doi:10.1186/s13068-017-0880-3)

**Figure S1**


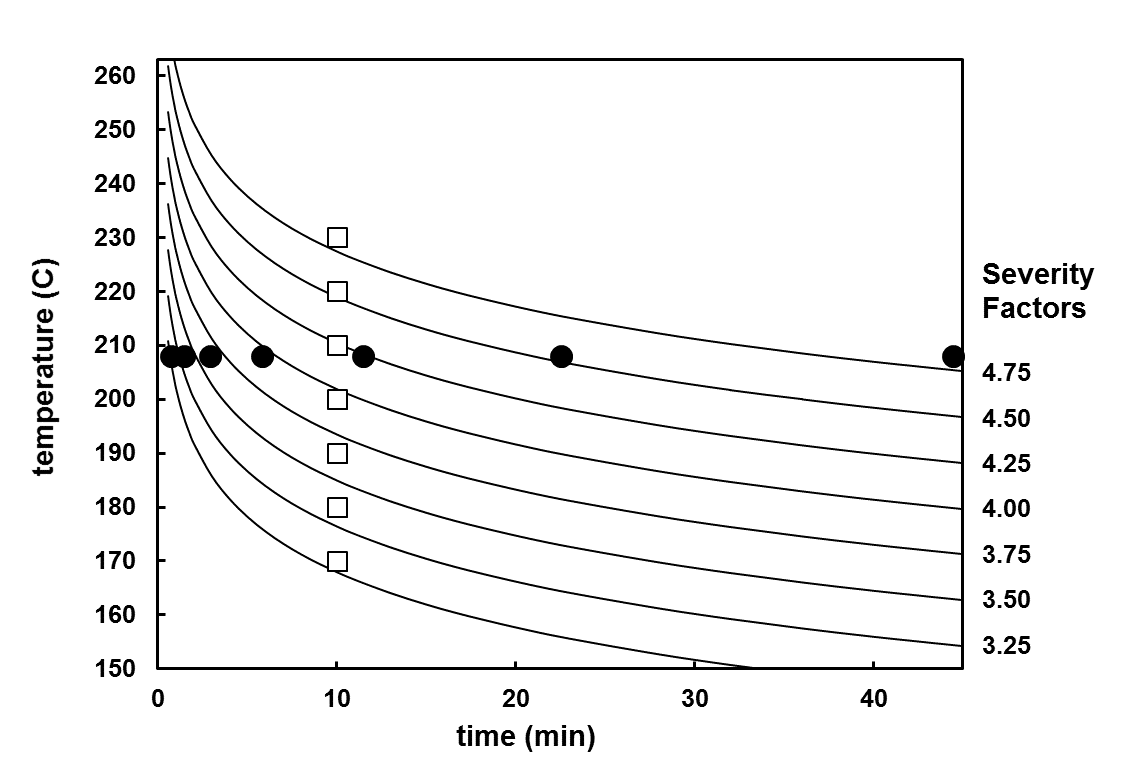

Supplement: Supplementary file 1 — Additional file 1. Pre-treatments at equivalent severities for steam treatments for 10 min and microwave treatments at 208 °C. [file 13068_2017_880_MOESM1_ESM.docx]

**Figure S3**


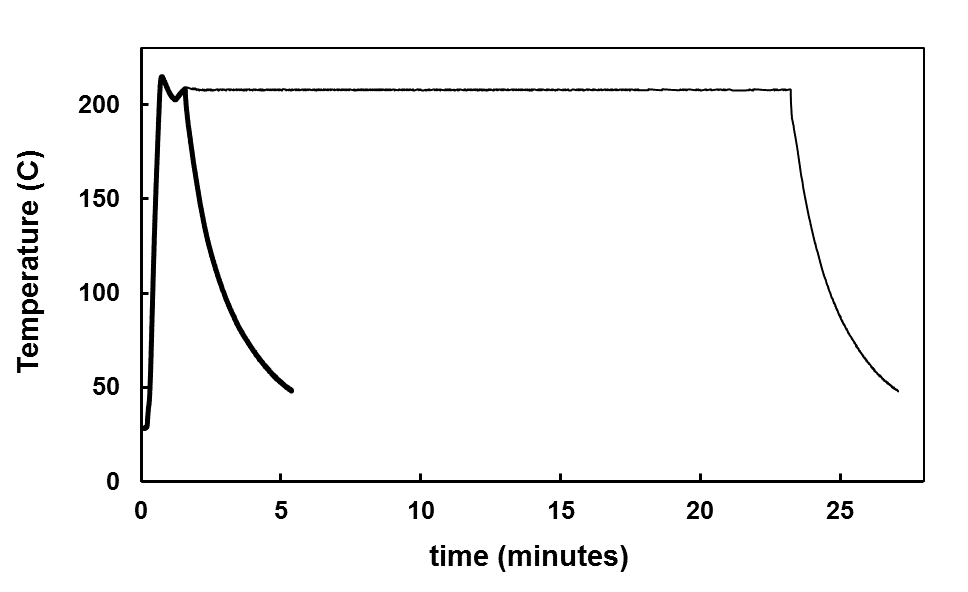

Supplement: Supplementary file 3 — Additional file 3. Temperature during short (bold trace) and long microwave treatments. The curves are coincident up to 1.81 min. [file 13068_2017_880_MOESM3_ESM.docx]
