# Supplementary material for: Bioethanol production from spent mushroom compost derived from chaff of millet and sorghum
Source: Biotechnol Biofuels. 2017 Aug 4;10:195. doi: 10.1186/s13068-017-0880-3 (PMC5545022; doi:10.1186/s13068-017-0880-3)
Supplement: Supplementary file 2 — Additional file 2. Table showing sample layout on plates. [file 13068_2017_880_MOESM2_ESM.docx]

|  |  |  | | 1 | 2 | 3 | 4 | 5 | 6 | 7 | 8 | 9 | 10 | 11 | 12 |
| --- | --- | --- | --- | --- | --- | --- | --- | --- | --- | --- | --- | --- | --- | --- | --- |
| Millet | KAN01 | | A | A1 | A2 | A3 | A4 | A5 | A6 | A7 | A8 | A9 | A10 | A11 | A12 |
| Millet | KAN02 | | B | B1 | B2 | B3 | B4 | B5 | B6 | B7 | B8 | B9 | B10 | B11 | B12 |
| Millet | KAN03 | | C | C1 | C2 | C3 | C4 | C5 | C6 | C7 | C8 | C9 | C10 | C11 | C12 |
| Sorghum | KAN04 | | D | D1 | D2 | D3 | D4 | D5 | D6 | D7 | D8 | D9 | D10 | D11 | D12 |
| Sorghum | KAB06 | | E | E1 | E2 | E3 | E4 | E5 | E6 | E7 | E8 | E9 | E10 | E11 | E12 |
| Sorghum | KAB07 | | F | F1 | F2 | F3 | F4 | F5 | F6 | F7 | F8 | F9 | F10 | F11 | F12 |
| Sorghum | KAB08 | | G | G1 | G2 | G3 | G4 | G5 | G6 | G7 | G8 | G9 | G10 | G11 | G12 |
|  | Blank | | H | H1 | H2 | H3 | H4 | H5 | H6 | H7 | H8 | H9 | H10 | H11 | H12 |
|  |  |  | | NCYC 2826 | NCYC 2826 | NCYC 3277 | NCYC 3277 | NCYC 3284 | NCYC 3284 | NCYC 3290 | NCYC 3290 | NCYC 3312 | NCYC 3312 | NCYC 3451 | NCYC 3451 |

**Figure S2**. The distribution of biomass and yeasts in a 96-well plate for SSF.
